# Stytra: An open-source, integrated system for stimulation, tracking and closed-loop behavioral experiments

**DOI:** 10.1371/journal.pcbi.1006699

**Published:** 2019-04-08

**Authors:** Vilim Štih, Luigi Petrucco, Andreas M. Kist, Ruben Portugues

**Affiliations:** Research Group of Sensorimotor Control, Max Planck Institute of Neurobiology, Martinsried, Germany; Radboud Universiteit Nijmegen, NETHERLANDS

## Abstract

We present Stytra, a flexible, open-source software package, written in Python and designed to cover all the general requirements involved in larval zebrafish behavioral experiments. It provides timed stimulus presentation, interfacing with external devices and simultaneous real-time tracking of behavioral parameters such as position, orientation, tail and eye motion in both freely-swimming and head-restrained preparations. Stytra logs all recorded quantities, metadata, and code version in standardized formats to allow full provenance tracking, from data acquisition through analysis to publication. The package is modular and expandable for different experimental protocols and setups. Current releases can be found at https://github.com/portugueslab/stytra. We also provide complete documentation with examples for extending the package to new stimuli and hardware, as well as a schema and parts list for behavioral setups. We showcase Stytra by reproducing previously published behavioral protocols in both head-restrained and freely-swimming larvae. We also demonstrate the use of the software in the context of a calcium imaging experiment, where it interfaces with other acquisition devices. Our aims are to enable more laboratories to easily implement behavioral experiments, as well as to provide a platform for sharing stimulus protocols that permits easy reproduction of experiments and straightforward validation. Finally, we demonstrate how Stytra can serve as a platform to design behavioral experiments involving tracking or visual stimulation with other animals and provide an example integration with the DeepLabCut neural network-based tracking method.

This is a *PLOS Computational Biology* Software paper.

## Introduction

The central goal of systems neuroscience is to explain the neural underpinnings of behavior. To investigate the link between sensory input, brain activity and animal behavior, relevant behavioral variables have to be recorded and quantified. Therefore, the same experimental paradigm has to be replicated in different experimental setups in order to combine it with different recording or stimulation techniques, and it needs to be reproducible across different laboratories. However, the setups generally rely on heterogeneous hardware and custom-made software tailored to the specific requirements of one experimental apparatus. Often, the code used is based on expensive software packages (such as LabView or Matlab), with open-source options for hardware control generally limited to one particular type or brand of devices. As a consequence, the same experimental protocol has to be implemented many times, thus wasting time and increasing potential sources of error. This makes sharing the code for replicating a scientific finding under the same experimental conditions very difficult.

To address these problems, we developed Stytra, a package that encompasses all the requirements of hardware control, stimulation and behavioral tracking that we encounter in our everyday experimental work. Our system, completely written in Python, provides a framework to assemble an experiment combining different input and output hardware and algorithms for online behavioral tracking and closed-loop stimulation. It is highly modular and can be extended to support new hardware devices or tracking algorithms. It facilitates reuse of different components of the package, encourages building upon existing work and enforces consistent data management. The definition of experimental protocols in high-level Python scripts makes it very suitable for version control and code sharing across laboratories, facilitating reproducibility and collaboration between scientists. Finally, it runs on all common desktop operating systems (Windows, MacOS and Linux), therefore incurring no additional costs on the software side. Similar approaches have already been made available for real-time tracking of zebrafish larvae [1, 2]. Still, to our knowledge, none of these solutions implement tracking functions for both head-restrained and freely-swimming larvae, they do not allow the use of custom tracking algorithms, and they do not provide a generic framework to design open- and closed-loop stimulation paradigms.

Stytra was developed primarily in the context of a laboratory working with larval zebrafish, and it fulfills the common requirements of behavioral paradigms used with this animal [3]: video tracking, visual stimulation and triggering of external devices. The tracking functions (for freely swimming and head-restrained fish) include both efficient re-implementations of published algorithms and newly-developed methods. Nevertheless, custom methods can easily be added. Common visual stimuli and methods for combining them and presenting them in different ways are provided. Our experimental setups are open-source as well [4]: hardware designs provided along with the documentation describe the apparatus required for performing common behavioral experiments in zebrafish in detail. The library provides many elements useful for designing behavioral experiments in Python, potentially offering a unified platform to build and share experiments in zebrafish neuroscience and behavioral research. We welcome and will support community contributions to expand the capabilities of the package to other paradigms and animals, although our development efforts will remain focused on zebrafish applications.

## Design and implementation

### Overview and library structure

We developed Stytra using the Python programming language. We endeavored to follow best practices in software engineering: separation of user interface and data processing code, modularity and consistent programming interfaces. In Stytra, new experiments can be designed using very simple Python syntax, allowing even beginners in programming to develop their own stimulation paradigms. Once defined, the experiment is controlled through a graphical user interface which can be used with no knowledge of Python. At the core of the Stytra package lies the Experiment object, which links all components that may be used in an experiment: stimulus presentation, camera, animal tracking, metadata and logging (S1 Fig).

**Fig 1:**
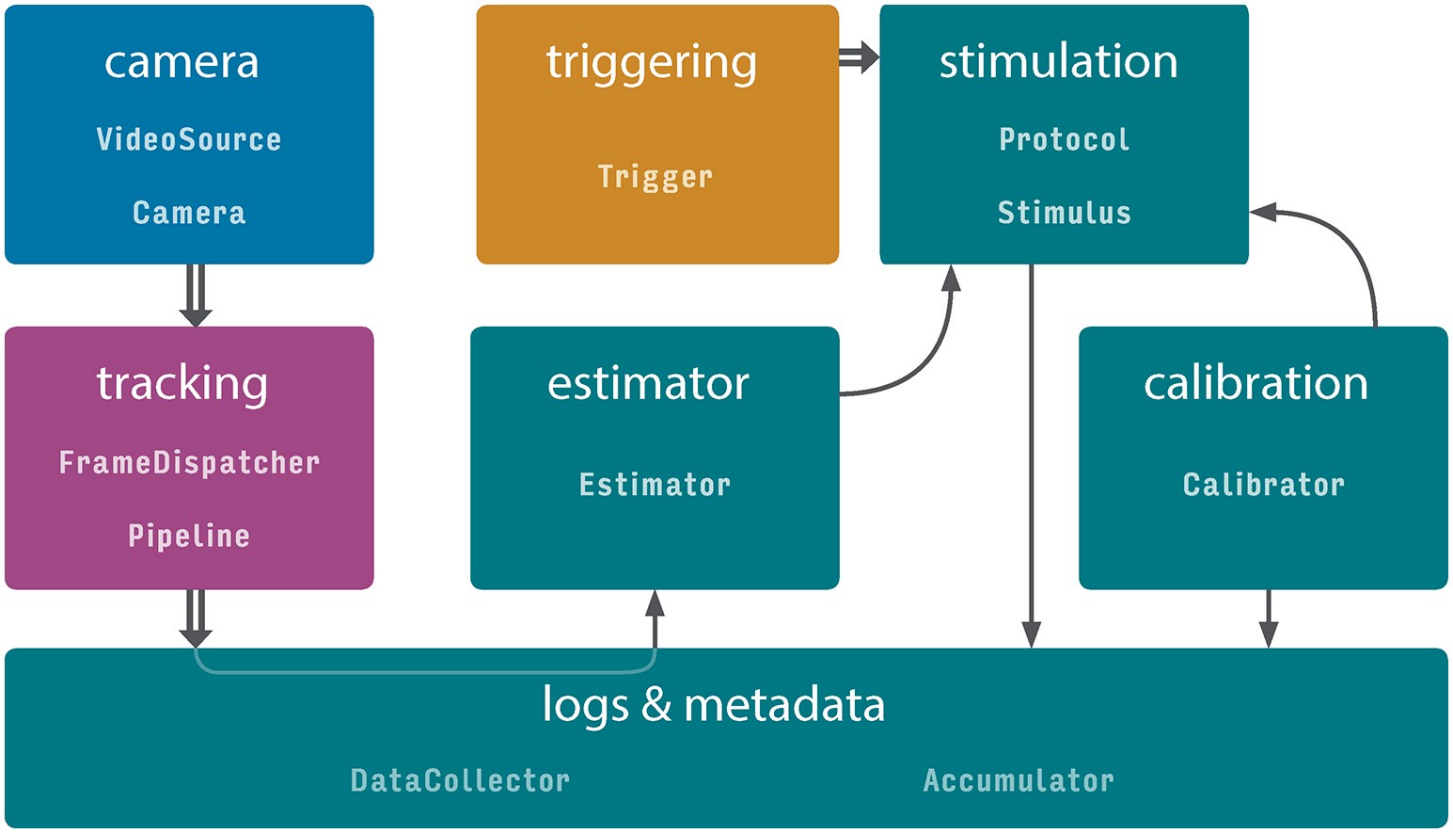
Data flow in Stytra. Communication between different parts of a Stytra experiment. Each color represents a separate process in which the module(s) are running. Data flow between modules within one process is depicted by arrows, and between processes as double arrows. The classes belonging to the data flow elements are displayed in monospace. A more comprehensive diagram of the classes is provided in S1 Fig. The user interface, the stimulus update and related functions such as the screen calibration and data saving are performed in the main process, colored in green. The stimulation can be triggered by a triggering process (in orange) that listens for an external triggering signal. Frames can be acquired from a camera process (in blue), analyzed by a tracking function (in purple), and the result can be streamed to the main process for data saving and used in closed-loop experiments via the estimator.

This organization enables composing different experimental paradigms with full code reuse. Improvement of different modules (e.g. the user interface, plotting or tracking) is therefore reflected in all experimental setups, and support for a new piece of hardware or tracking function can be added with minimal effort and interference with other parts of the project. Online image processing is organized along a sequence of steps: first, images are acquired from the camera, then the image is filtered and tracked, and the tracking results are saved. Acquisition, tracking and data saving occur in separate processes (depicted in blue, purple, and green in Fig 1). This approach improves the reliability and the performance of online behavioral tracking, and exploits the advantages of multi-core processors. After processing, streaming numerical data (such as tracking results and dynamic parameters of stimuli) is passed into data accumulators in the main thread, and a user-selected subset can be plotted in real time and saved in one of the several supported formats. Moreover, for every experimental session all changeable properties impacting the execution of the experiment are recorded and saved. Finally, as the software package is version-controlled, the version (commit hash) of the software in use is saved as well, ensuring the complete reproducibility of every experiment.

### Building and running an experiment in Stytra

**Fig 2:**
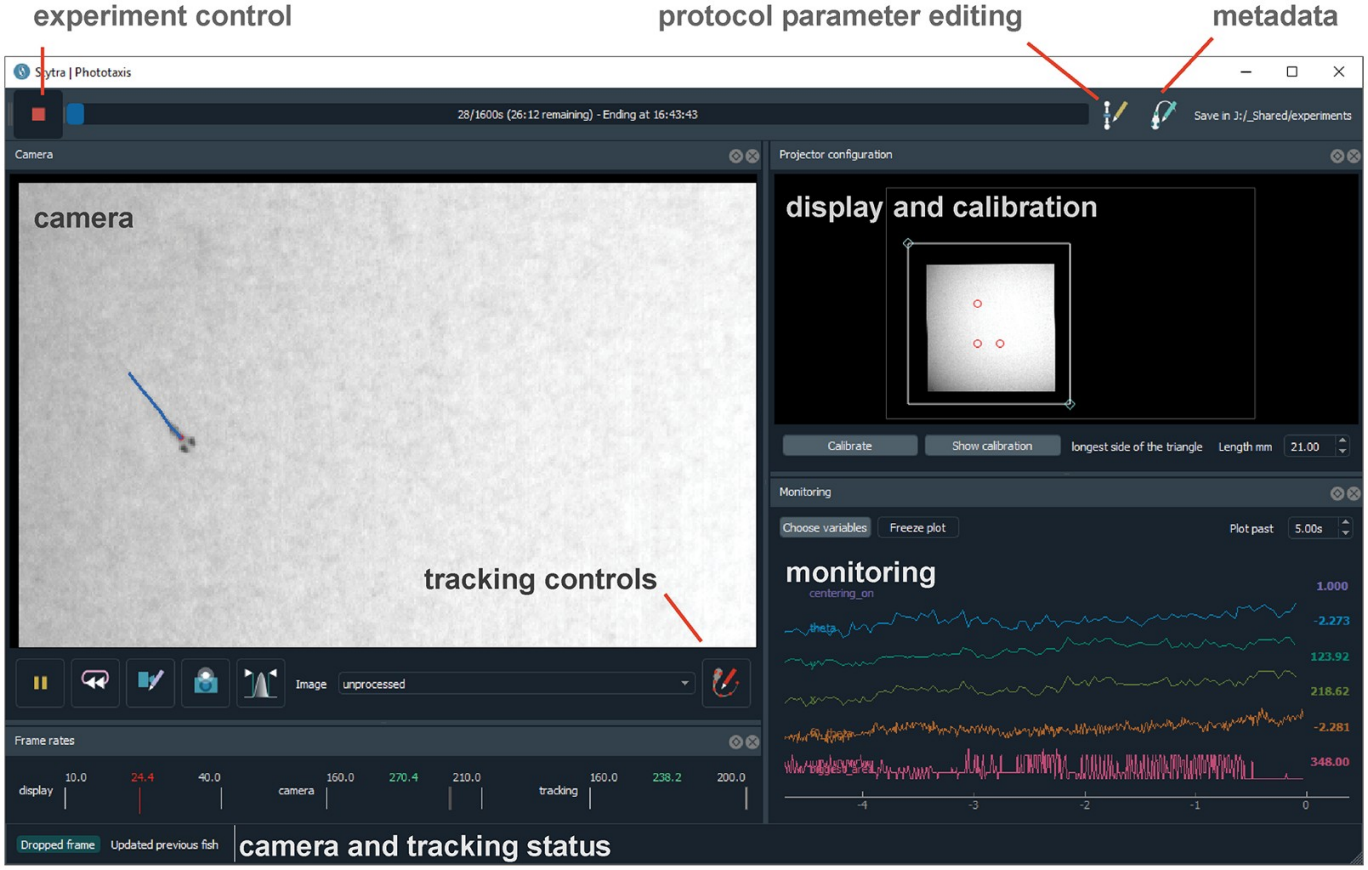
Screen capture of the software in use. The various behavioral paradigms supported by Stytra provide the user with a consistent interface to control experiments. The toolbar on top controls aspects of running the experiment, a camera panel shows the tracking results superimposed on the camera image, a calibration panel enables quick positioning and calibration of the stimulus display and a monitoring panel plots a user-selected subset of experimental variables.

The Experiment object binds all the different components required to run an experiment. The most basic Experiment object performs the presentation of a succession of stimuli, saving the experiment metadata and the stimulation log. For experiments including video tracking, the TrackingExperiment object augments the basic Experiment with features such as camera frame acquisitions and online image analysis. The image analysis pipeline can be one of the zebrafish specific pipelines supplied with Stytra, or a custom tracking pipeline. The Experiment is linked to the user interface for controlling the running of stimulation protocols, inserting metadata, controlling parameters, and calibrating the stimulus display (Fig 2). In general, the users do not need to define new types of Experiment objects for every new experimental paradigm. Instead, paradigms are implemented by defining a Protocol object which contains the stimulus sequence (as described below) and a configuration dictionary with information about the camera, tracking pipeline, and triggering. The appropriate Experiment object can be automatically instantiated from the configuration dictionary using the Stytra constructor. Alternatively, an Experiment can be instantiated and run from the experiment script, as described in the documentation examples. Ideally, the provided Experiment objects should cover most of the requirements of zebrafish behavioral experiments, and redefining the Experiment is required only if one needs to modify the graphical user interface (gui), add more nodes in the data pipeline (screens or cameras) or implement more specific customizations. A more detailed depiction of the connections and versions of different objects is depicted in S1 Fig. For examples of how to create a Protocol and run experiments in Stytra, see the Usage examples box and the more detailed examples gallery in the documentation.

### Stimulus design

Experimental protocols in Stytra are defined as sequences of timed stimuli presented to the animal through a projector or external actuators. A sequence of stimuli, defined as a Python list of Stimulus objects, is defined in a Protocol object (see Usage examples box). This structure enables straightforward design of new experimental protocols, requiring very little knowledge of the general structure of the library and only basic Python syntax. A dedicated class coordinates the timed execution of the protocol relying on a QTimer from the PyQt5 library, ensuring a temporal resolution in the order of 15-20 ms (around the response time of a normal monitor, see S2 Fig). Drawing very complex stimuli consisting of many polygons or requiring online computation of large arrays can decrease the stimulus display performance. The stimulus display framerate can be monitored online from the user interface when the protocol is running (see the lower left corner of the window in Fig 2). Milli- or microsecond precision, which might be required for optogenetic experiments, for example, is currently not supported. Each Stimulus has methods which are called at starting time or at every subsequent time step while it is set. In this way one can generate dynamically changing stimuli, or trigger external devices. New Stimulus types can be easily added to the library just by subclassing Stimulus and re-defining the Stimulus.start() and Stimulus.update() methods.

A large number of stimuli is included in the package. In particular, a library of visual stimuli has been implemented as VisualStimulus objects using the QPainter object, a part of the Qt gui library, enabling efficient drawing with OpenGL. Relying on a set of high-level drawing primitives makes the code very readable and maintainable. Stytra already includes common stimuli used in visual neuroscience, such as moving bars, dots, whole-field translation or rotations of patterns on a screen, and additional features such as movie playback and the presentation of images from a file (which can be generated by packages such as Imagen [5]). The classes describing visual stimuli can be combined, and new stimuli where these patterns are moved or masked can be quickly defined by combining the appropriate Stimulus types. Finally, new stimuli can be easily created by redefining the paint() method in a new VisualStimulus object. Multiple stimuli can be presented simultaneously using StimulusCombiner. Presenting different stimuli depending on animal behavior or external signals can be achieved using the ConditionalStimulus container, or with similarly designed custom objects. Visual stimuli are usually displayed on a secondary screen, therefore Stytra provides a convenient interface for positioning and calibrating the stimulation window (visible in Fig 2 on the right-hand side). Although in our experiments we are using a single stimulation monitor, displaying stimuli on multiple screens can be achieved with virtual desktop technology or screen-splitting hardware boards. Importantly, all stimulus parameters are specified in physical units and are therefore independent of the display hardware. Finally, the timed execution of code inside Stimulus objects can be used to control hardware via I/O boards or serial communication with micro-controllers such as Arduino or MicroPython PyBoard. For example, in this way one may deliver odors or temperature stimuli or optogenetic stimulation. Examples for all these kinds of stimuli are provided in the main repository.

### Usage examples

Here we present the main parts of simple scripts that can be used to run a Stytra experiment. The complete scripts can be found in the Stytra repository under stytra/examples. Stytra is run in most cases by defining a stimulus sequence in a Protocol object. This custom protocol is passed to the Stytra constructor, which creates an appropriate Experiment object. The subclass of Experiment is selected depending on the configuration passed through either the Stytra constructor or the stytra_config attribute of the Protocol. The online documentation contains an example of how to use a custom Experiment class.

#### Creating and running a protocol

To create an experiment, a Protocol class has to be defined. The Protocol.get_stim_sequence() method returns the sequence of stimuli that will be presented in the experiment. A Protocol object is then passed as an argument to the instance of Stytra that will run it.

Example:

from stytra import Stytra, Protocol

from stytra.stimulation.stimuli import Pause, FullFieldVisualStimulus

class FlashProtocol(Protocol):

 name = “flash protocol” # protocol name

 def get_stim_sequence(self):

  stimuli = [Pause(duration = 9), # black screen, 9 sec FullFieldVisualStimulus(duration = 1, # flash, 1 sec color = (255, 255, 255))]

  return stimuli

Stytra(protocol = FlashProtocol())

#### Creating a new stimulus

In an experiment it might be necessary to use a stimulus type not available in the existing library. To design a new stimulus, a Stimulus subclass has to be created and its Stimulus.start() and Stimulus.update() methods should be overwritten. In the following piece of code, we create a closed-loop stimulus which turns the screen red when the fish is swimming. To achieve this, we redefine the Stimulus.update() to change the color attribute, and the Stimulus.paint() to paint the screen red. The stytra_config attribute defines the video source (a Ximea camera), and the tracking functions (tail tracking with vigor as a velocity estimator):

from stytra import Stytra, Protocol

from stytra.stimulation.stimuli import VisualStimulus

from PyQt5.QtCore import QRect

from PyQt5.QtGui import QBrush, QColor

class NewStimulus(VisualStimulus):

 def _ _init_ _(self, *args, **kwargs):

  super()._ _init_ _(*args, **kwargs)

  self.color = (255, 255, 255)

 def paint(self, painter, w, h):

  # painter, w and h come from the Qt library drawing functions.

  # painter: QPainter object;

  # w, h: width and height of the window

  painter.setBrush(QBrush(QColor(*self.color))) # Use chosen color

  painter.drawRect(QRect(0, 0, w, h)) # draw full field rectangle

 def update(self):

  fish_vel = self._experiment.estimator.get_velocity()

  # change color if speed of the fish is higher than threshold:

  if fish_vel < -15:

   self.color = (255, 0, 0)

  else:

   self.color = (255, 255, 255)

class CustomProtocol(Protocol):

 name = “custom protocol” # protocol name

 # Here we define tracking method, vigor estimator, and add a camera:

 stytra_config = dict(tracking = dict(method = “tail”, estimator = “vigor”), camera = “ximea”)

 def get_stim_sequence(self):

  return [NewStimulus(duration = 10)]

Stytra(protocol = CustomProtocol())

### Image acquisition and tracking

#### Image acquisition

A key feature of Stytra is the extraction of relevant behavioral features in real time from video inputs. The Camera object provides an interface for grabbing frames and setting parameters for a range of different camera types. Currently supported models include those by XIMEA, AVT, PointGray/FLIR, and Mikrotron, as well as webcams supported by OpenCV [6]. Support for other cameras can be added as long as a Python or C api exists. In addition, previously-recorded videos can also be processed, allowing for offline tracking. Frames are acquired from the original source in a process separated from the user interface and stimulus display. This ensures that the acquisition and tracking frame rate are independent of the stimulus display, which, depending on the complexity of the stimulus and output resolution, can be between 30 and 60 Hz.

#### Tracking pipelines

The tracking process receives acquired frames and handles animal tracking (represented in Fig 1). Image processing and tracking are defined in subclasses of Pipeline objects and contain a tree of processing nodes, starting from input images and ending with tracking nodes that take images as input and give tracking results as output. This structure allows for multiple tracking functions to be applied on the same input image(s). Currently implemented image processing nodes include image filtering (down-sampling, inversion and low-pass filtering) and background subtraction. The outputs of the tracking nodes are assembled together and streamed to the main process, where the data is saved and visualized. The Pipeline object also allows specifying a custom camera overlay to display the results of the tracking and an additional plotting widget for an alternative visualization of data. This modular structure allows easy expansion of the library: new functions for pre-filtering or tracking can be incorporated into the pipeline with minimal effort. Pipelines to track tail and eye position in head-restrained fish, as well as fish position and orientation in an open arena, are included in Stytra. Parts of the tracking functions use the OpenCV computer vision library. Time-critical functions are compiled with the Numba library to increase their performance.

#### Behavior tracking in head-restrained fish

**Fig 3:**
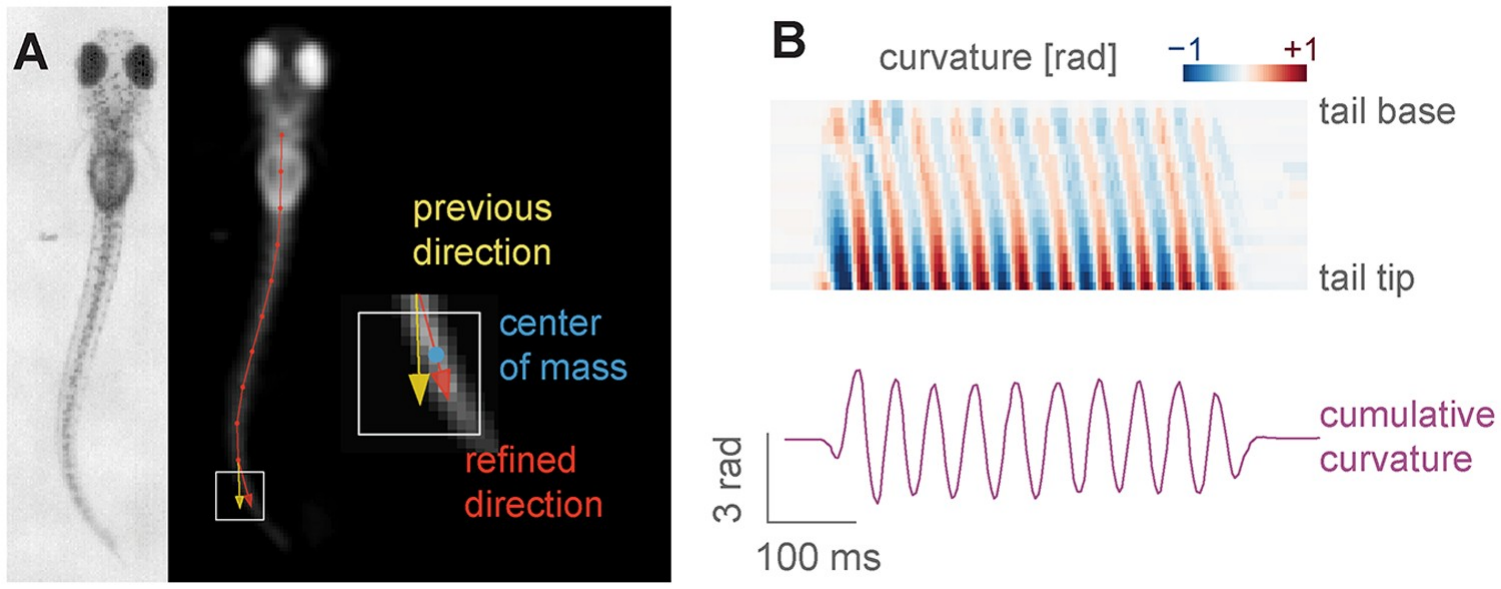
Head-restrained tail tracking in Stytra. A) The image is first pre-processed by inverting, down-scaling, blurring and clipping, resulting in the image on the right, where the fish is the only object brighter than the background. Then, tail tracing starts from a user-defined point, and in the direction determined by another user-defined point at the end of the tail at rest. For each segment, a square (outlined in white) in the direction of the previous segment (yellow) is sampled, and the direction for the next segment is chosen as the vector (red) connecting the previous segment end and the center of mass of the sampled square (blue). B) A heatmap showing the angles of the tail segments from the start to the end of the tail during a bout, and a trace representing the cumulative curvature sum from a behaving animal. The total curvature is just the difference in angle between the first and last tail segment (adding up angle differences between all segments, only these two terms remain).

***Tail tracking***. Zebrafish larvae swim in discrete units called bouts, and different types of swim bouts, from startle responses to forward swimming are caused by different tail motion patterns [7]. The tail of the larvae can be easily skeletonized and described as a curve discretized into 7-10 segments [8] (Fig 3A). The tail tracking functions work by finding the angle of a tail segment given the position and the orientation of the previous one. The starting position of the tail, as well as a rough tail orientation and length need to be specified beforehand using start and end points, movable over the camera image displayed in the user interface (as can be seen in Fig 3A).

To find the tail segments, two different functions are implemented. The first one looks at pixels along an arc to find their maximum (or minimum, if the image is inverted) where the current segment would end (as already described in e.g. [8]). The second method, introduced here, is based on centers of mass of sampling windows (Fig 3), and provides a more reliable and smoother estimate over a wider range of resolutions and illumination methods. The image contrast and tail segment numbers have to be adjusted for each setup, which can be easily accomplished through the live view of the filtering and tracking results. In the documentation we provide guidelines on choosing these parameters. To compare results across different setups which might have different camera resolutions, the resulting tail shape can be interpolated to a fixed number of segments regardless of the number of traced points.

**Fig 4:**
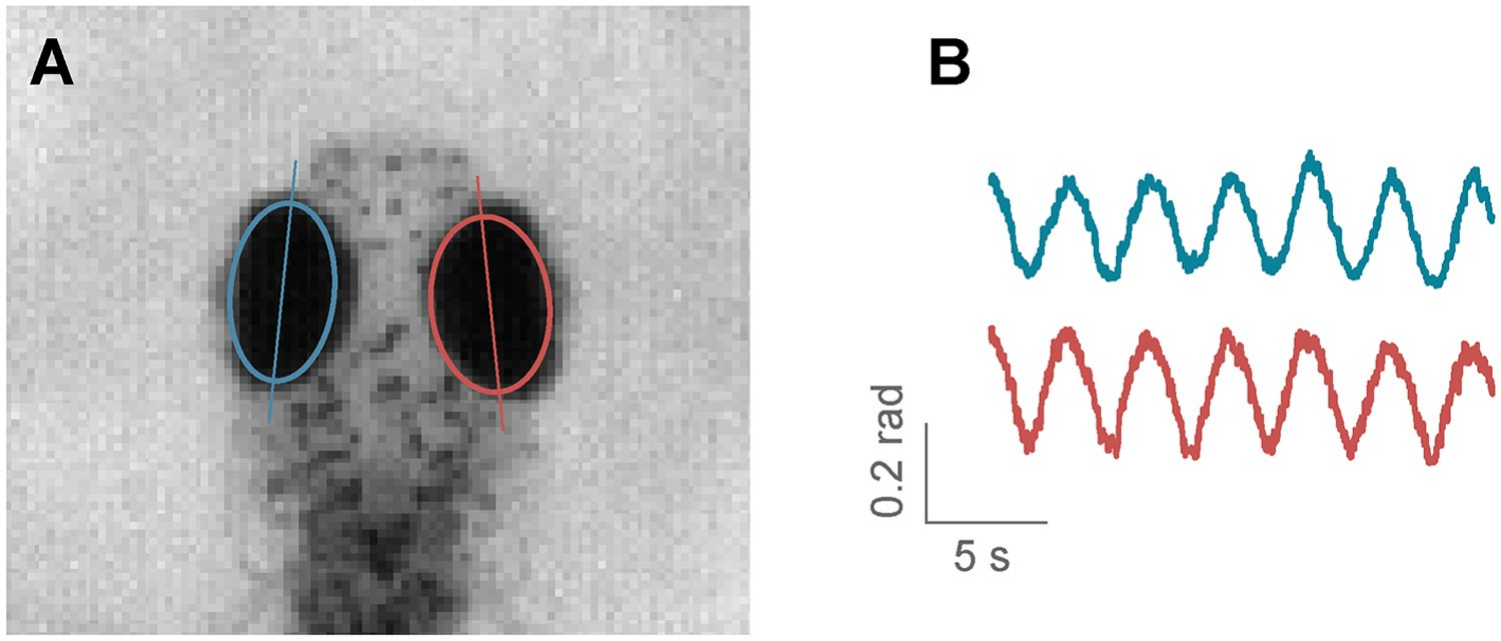
Eye tracking in Stytra. A) Eyes are detected by fitting an ellipse to the connected components of the image of the fish head after thresholding. B) Example trace of eye motion in response to a full-field rotating background.

***Eye tracking***. Zebrafish larvae move their eyes to stabilize their gaze in response to whole field motion, perform re-positioning saccades, and converge their eyes to follow a potential prey in hunting maneuvers [9]. Naso-temporal eye movements can be described by the eye orientation with respect to the fish axis. Given the ellipsoidal shape of the eyes when seen from above, to find their orientation it is sufficient to fit an ellipse to the eye pixels and determine the angle of the major axis [9]. In Stytra, a movable and scalable rectangular region can be used to select the area of the camera view containing the eyes. As eyes are usually much darker than the background, with proper illumination conditions it is sufficient to binarize the image with an adjustable threshold which selects the pixels belonging to the eyes. Then, functions from the OpenCV library are used to find the two largest connected components of the binarized region and fits an ellipse to them. The absolute angle of the major axis of the ellipse is recorded as the eye angle (Fig 4). A live preview of the binarized image and the extracted ellipses helps the user to adjust the parameters.

#### Freely-swimming fish tracking

**Fig 5:**
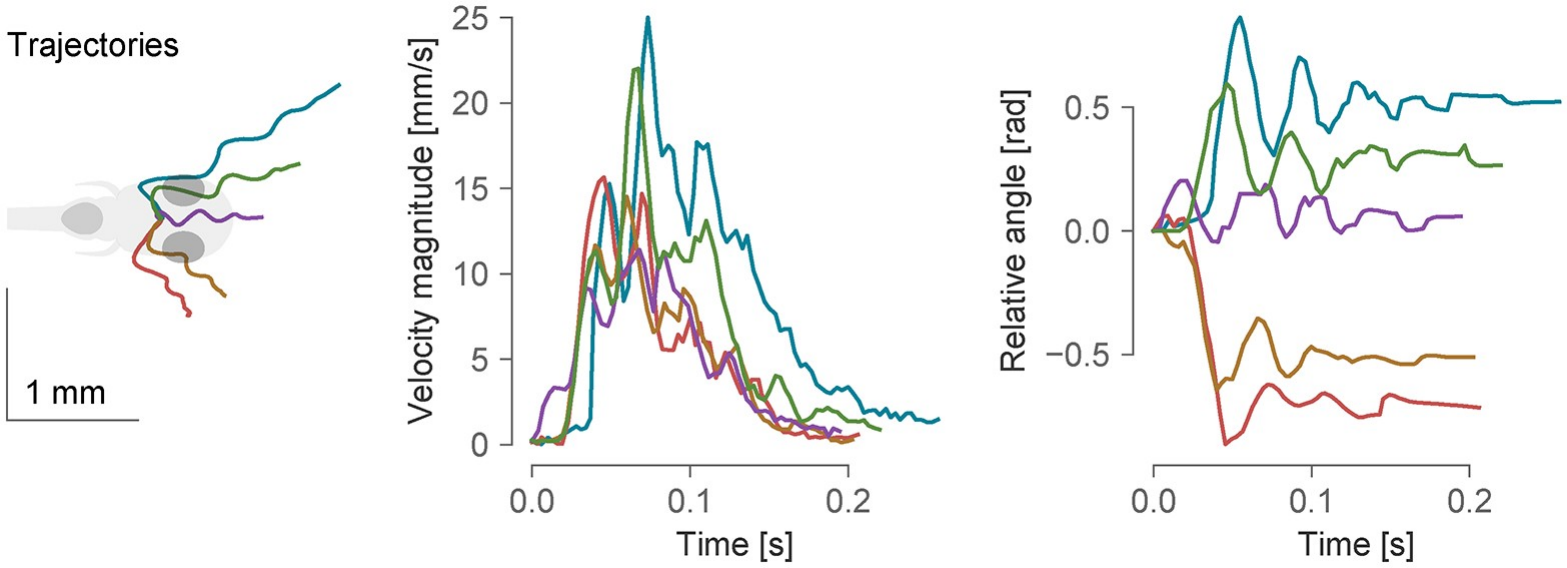
Example bouts tracked from freely-swimming fish. From left to right: trajectories of bouts in different directions, the velocity magnitude and the total angle change during the course of the bouts. In the left-most panel, all trajectories were realigned such that the initial position and orientation of the fish were the same. The data was sampled at 300 Hz.

To support different kinds of paradigms where fish are not head-restrained, we provide functions for freely-swimming fish tracking. The range of behavioral paradigms include investigating movement evoked by different kinds of stimuli, characterizing motion kinematics and assessing consequences of pharmacological or genetic interventions. To track the fish in an open arena, the first required step is background subtraction. The background is modelled with a mean image taken from multiple frames averaged in time, and slowly updated with an adjustable time constant. The subsequently processed image is the negative difference between the current frame and the threshold (pixels that are darker than the background are active). This image is first thresholded and regions within the right area range are found. Both eyes and the swim bladder are found as darker parts inside of these regions, and the center of mass of the three objects (two eyes and swim bladder) is taken as the center of the fish head. The direction of the tail is found by searching for the point with the largest difference from the background on a circle of half-tail radius. This direction is subsequently refined in the course of tail tracking, as described in the tail tracking section. The kinematic parameters are smoothed by Kalman filtering. An example resulting from tracking multiple fish simultaneously is shown in Fig 5. Fish identities are maintained constant while they are in the field of view and not overlapping, by keeping track of the previous positions and orientations. The number of fish does not significantly impact performance, however the resolution of the camera does, so we recommend a well-configured modern computer (7th generation and above Intel Core i7 processors or amd Ryzen) for tracking multiple fish in a 90 mm dish. In our experiments not more than 3 fish are usually present, and a tracking framerate of 300 Hz can be reached reliably. We have also tracked individual fish in a 24-well plate, which presented no performance issues at 100 Hz with a mid-range cpu. Simpler tracking scenarios for screening, where the exact position, orientation and tail curvature of individuals are not of interest, can work with even higher numbers of animals.

For closed-loop experiments, the camera view and the projected area need to be aligned to lock the stimulus to the fish position. To this end, a calibration module inside of Stytra finds the mapping between the area covered by the camera and the area illuminated by the screen. During calibration, three points are projected on the screen and detected as local maxima on the camera image. Then, a transformation matrix is computed to align the projected and recorded points. If the setup elements are kept firmly in place, the calibration has to be done only once, although regular checking of the calibration on a regular basis is encouraged.

#### Custom tracking functions

**Fig 6:**
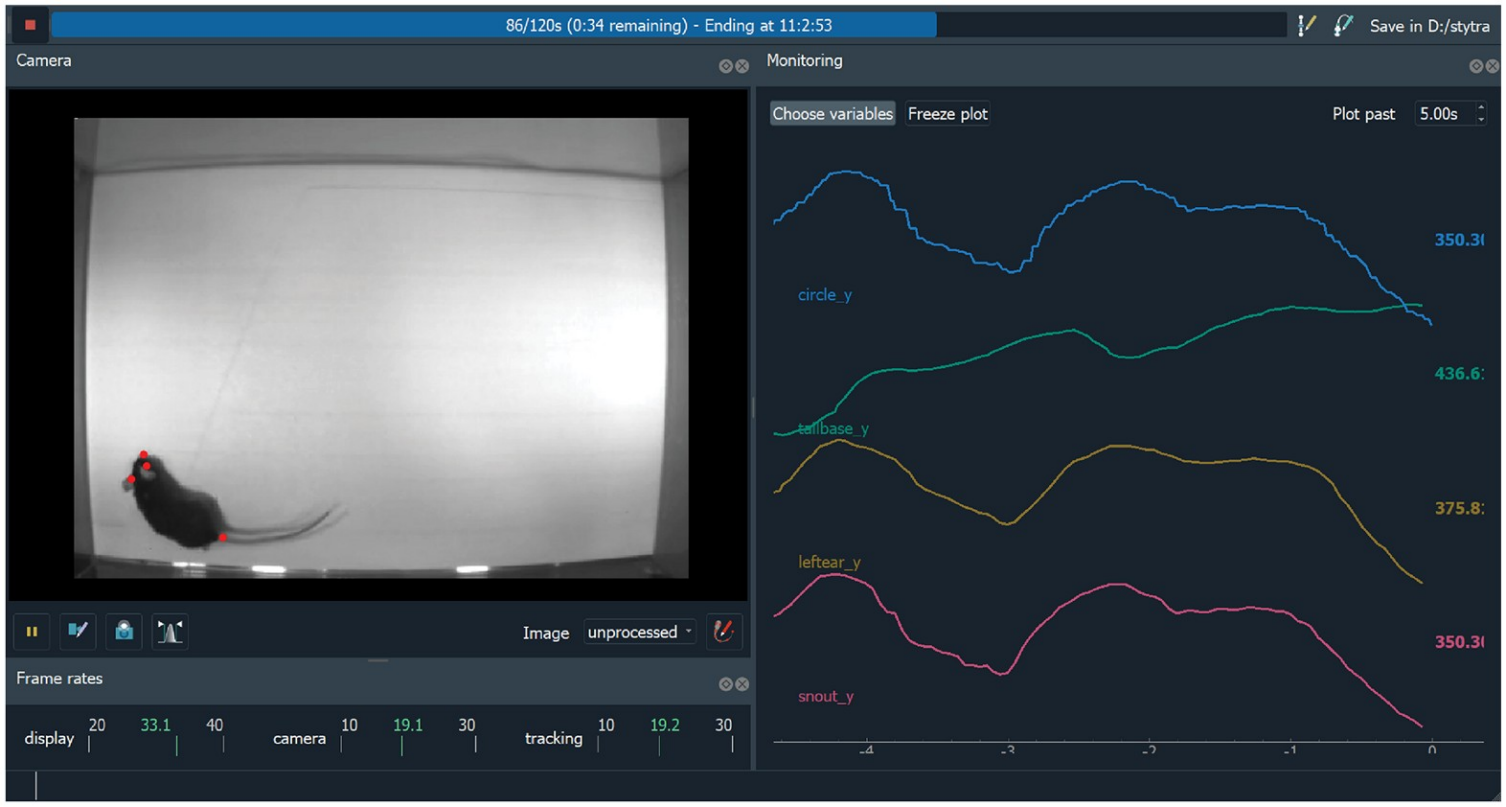
Screenshot of DeepLabCut-based rat tracking in Stytra. On the left, the 4 detected keypoints (snout, two ears and tail base) in red are superimposed on the video. On the right, traces tracking the coordinates of the animal are displayed, along with a parameter of of a closed-loop stimulus (a circle that would be tracking a rat). The video displayed was provided with the DeepLabCut repository [10].

Stytra is designed in an extensible fashion and video tracking algorithms for other animals can be easily added. To demonstrate this, we provide a small example of DeepLabCut-based tracking, which can be integrated with very few lines of code and immediately used with closed-loop stimuli. DeepLabCut is a convolutional neural network-based pose estimation toolbox [10] built on top of the DeeperCut architecture [11]. We incorporated an open-field recording example with the video and parameters provided in the original repository (see Fig 6). The code for this example is in a separate GitHub repository, listed at the end of the manuscript. The tracking performance of DeepLabCut mainly depends on video resolution and cpu and gpu performance. We managed to obtain a tracking speed of 20 Hz (resulting in a tracking latency of 50 ms) for a 640x480 px video on a computer equipped with a nVidia GeForce gtx Titan X gpu and Intel Xeon E5-2687W v3 cpu. For a detailed investigation of DeepLabCut performance see [12].

### Closed-loop stimuli design

**Fig 7:**
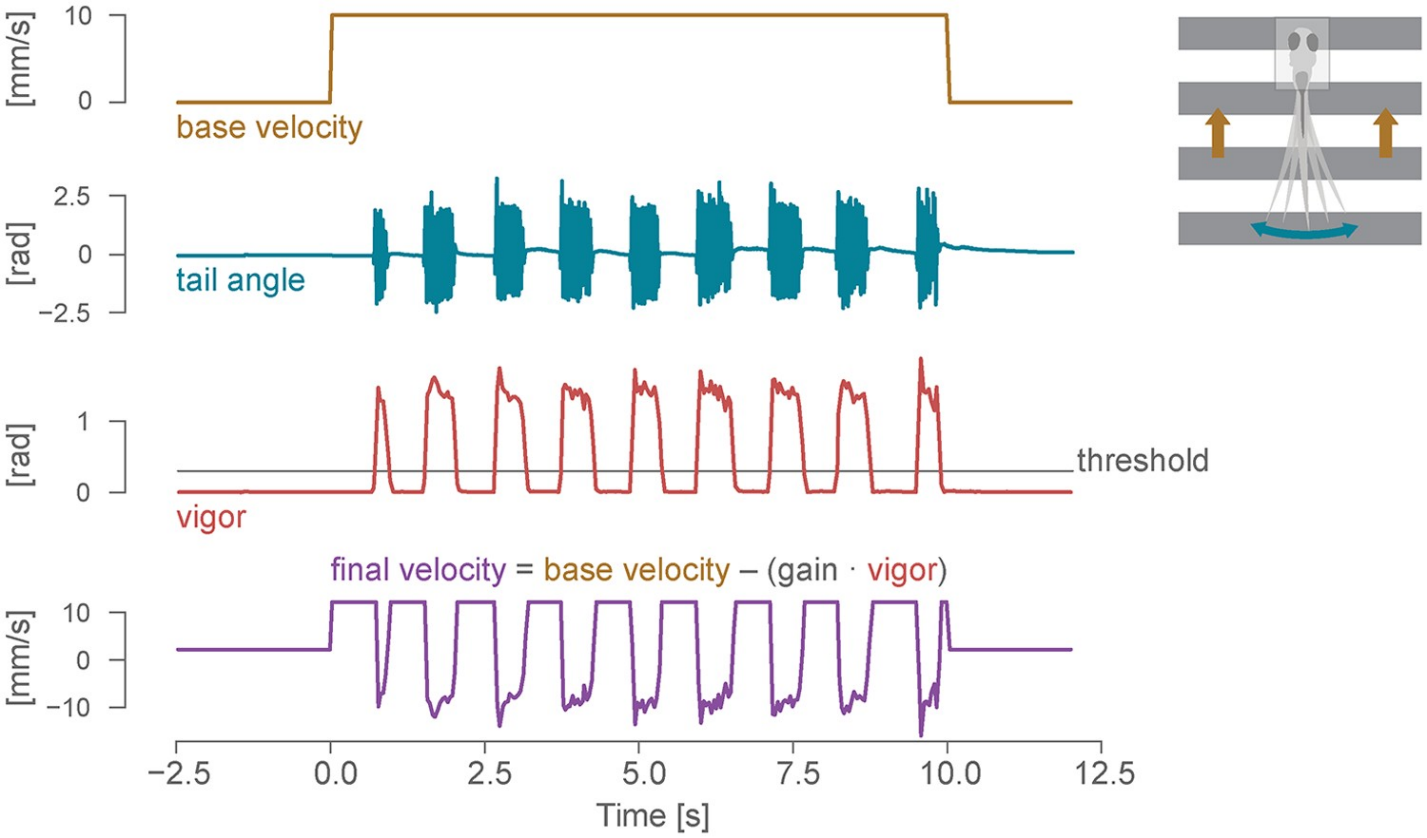
Closed-loop optomotor assay. Dynamic update of the stimulus in a closed-loop assay for the optomotor response. From top: open-loop velocity of the gratings moving caudo-rostrally below the fish; cumulative tail angle (see the tail tracking section and Fig 3 for details); bout vigor, estimated by calculating the instantaneous standard deviation of the angle sum in a 50 ms window; final closed-loop velocity of the gratings, with backward movements induced by the fish swimming.

Stimuli whose state depends on the behavior of the fish (position and orientation for freely swimming fish, and tail or eye motion for head-restrained fish) are controlled by linking the behavioral state logs to the stimulus display via Estimator objects (see Fig 1). An Estimator receives a data stream from a tracking function (such as tail angles), and uses it together with calibration parameters to estimate some quantity online. For example, a good proxy for fish velocity is the standard deviation of the tail curvature over a window of 50 ms [13], which we refer to as vigor. Fig 7 shows an example of how vigor can be used in a closed-loop optomotor assay. When presented with a global motion of the visual field in the caudal-rostral direction, the fish tend to swim in the direction of perceived motion to minimize the visual flow, a reflex known as the optomotor response [3, 14]. The visual feedback during the swimming bout is a crucial cue that the larvae use to control their movements. In this closed-loop experiment, we use the vigor-based estimation of fish forward velocity, together with a gain factor, to dynamically adjust the velocity of the gratings to match the visual flow expected by a forward swimming fish. The gain parameter can be changed to experimentally manipulate the speed of the visual feedback received by the larvae [13] (see below).

Closed-loop stimuli may be important for freely swimming fish as well, for example to display patterns or motion which always maintain the same spatial relationship to the swimming fish by matching the stimulus location and orientation to that of the fish.

### Synchronization with external devices

Stytra is designed to support the presentation of stimuli that need to be synchronized with a separate acquisition program, e.g. for calcium imaging or electrophysiology. To this end, the Trigger object enables communication with external devices and different computers to synchronize the beginning of the experiment. The Trigger object runs in a separate process, ensuring that the interface is not blocked while waiting for trigger signals, and it can be used to either trigger the beginning of the experiment, or to trigger arbitrary parts of the protocol using the existing TriggerStimulus object or similar custom stimuli. Two ways of receiving the triggering signal are already supported in the library: ttl pulse triggering via a LabJack board, and communication over a local network employing the ZeroMQ library. Messages exchanged through ZeroMQ can also contain data, such as the microscope configuration, that will be saved together with the rest of the experiment metadata. The triggering module is designed to be easily expandable, and we provide instructions for writing custom trigger objects. In our lab the two-photon microscope is controlled by custom LabView software, which we extended to include ZeroMQ communication with Stytra. An example LabView program that can be used to trigger Stytra is illustrated in the triggering section of the documentation. In Results, we describe an example experiment using this triggering configuration to link behavioral and stimulus quantities and the recorded calcium responses. Proprietary scanning programs where this cannot be achieved can still trigger Stytra using ttl pulses.

### Data collection

The design of Stytra encourages automatic data management. A dedicated DataCollector object is used to log the metadata about the experiment. Parameters from the entire program are appended to a single hierarchical parameter tree, which is saved at the end of the experiment. Quantities in the tree can come from different sources. Firstly, parameters can be added at any point in the code. For example, at every run the current version number of Stytra and git commit are detected and saved, together with the versions of the dependencies. Secondly, many of the key objects of Stytra (tracking nodes, display and camera controllers…) are parametrized though a custom parameters package (lightparam). When constructing them, one needs to pass the parameter tree that collects the data. This ensures that all quantities needed to replicate the experiment are collected within the metadata file. Finally, dedicated parametrized objects can be used to manually input metadata concerning the animal (age, genotype, etc.) or the experiment (setup, session, etc.). These classes can be customized to automatically include lab-specific metadata options, such as setup identifiers or animal lines (examples for this customization are provided in the documentation). Various logs accompanying the experiment run (state of the stimuli, the raw tracking variables and the estimated state of the fish) are saved as tabular data. The supported data formats are CSV, HDF5 and Feather, but others could be added as long as they provide an interface to the Pandas library. To demonstrate the convenience of the data and metadata saving methods of Stytra, we made example data available together with Jupyter notebooks for the analyses that can reproduce the figures in this paper. Finally, a central experiment database can be connected to keep track of all the experiments in a lab or institute. The documentation provides an example of a MongoDB database connection.

### Setup hardware

**Fig 8:**
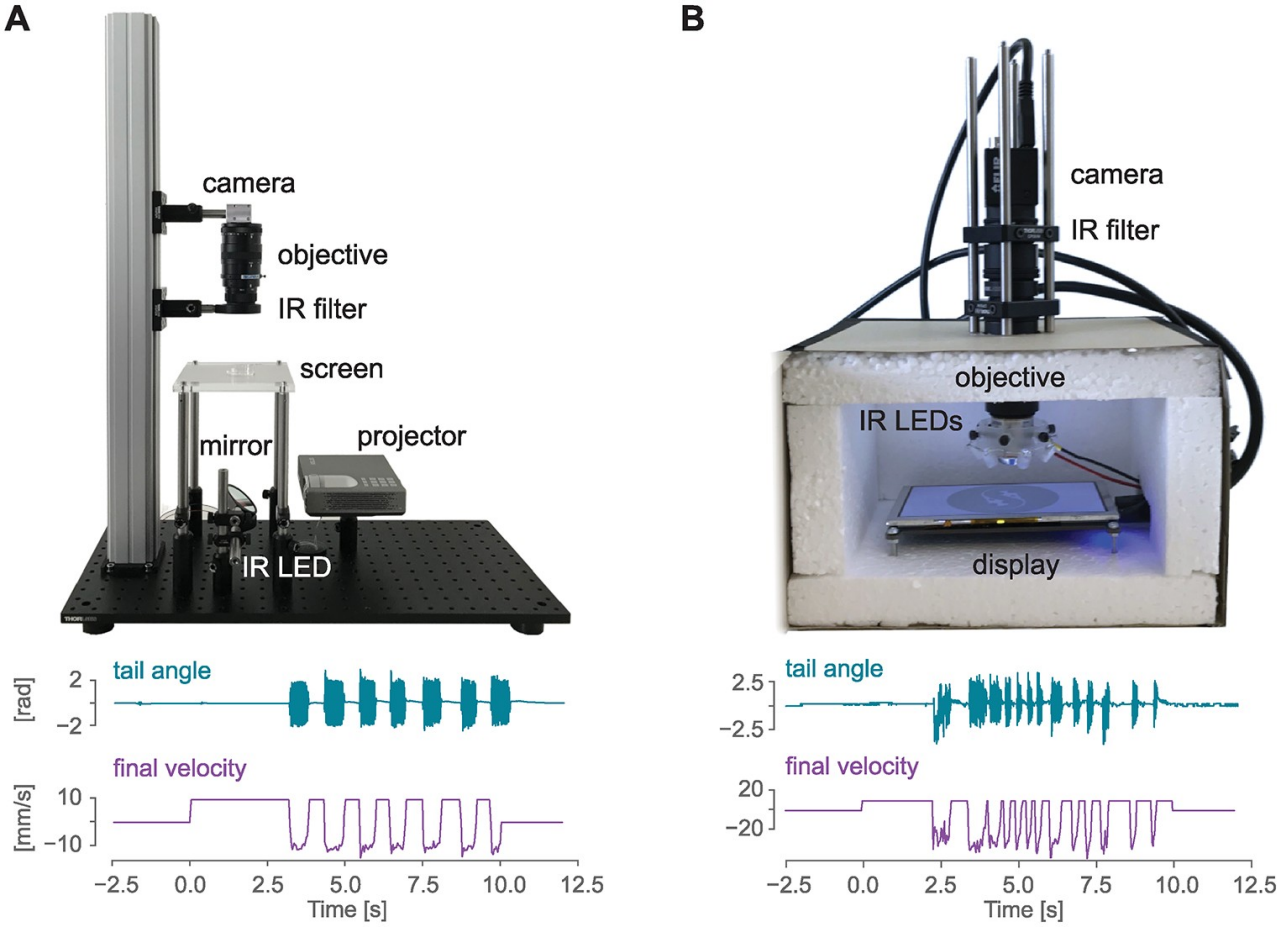
Hardware for zebrafish behavior experiments. A) Above: sample image of a behavioral setup that can be used to track head-restrained zebrafish tail end eyes (the opaque enclosure has been removed for visualization purposes). Below: sample traces for tail angle and grating velocity obtained from this setup with the closed-loop experiment described in Fig 7. B) A low-cost version of the setup presented in A) that can be used to investigate behavior in the head-restrained fish, and sample traces from this setup. A detailed description of the setup together with a complete list of parts can be found at www.portugueslab.com/stytra/hardware_list.

In our effort to make experiments as open and reproducible as possible, we documented example setups that can be used together with the Stytra software for performing behavioral experiments in head-restrained and freely swimming fish (Fig 8). In general, the minimal setup for tracking the fish larvae requires a high-speed camera (a minimum of 100 Hz is required to capture the most common tail beats which have a frequency up to 50 Hz, but we recommend at least 300 Hz to describe the details of the tail kinematics). The camera must be equipped with a suitable objective: a macro lens for the head-restrained tail tracking or a normal lens for the freely swimming recordings, where a smaller magnification and a larger field of view are required. More detailed camera and lens guidelines can be found in the documentation. Infrared illumination is then used to provide contrast without interfering with the animal’s visual perception. Since fish strongly rely on vision and many of their reflexes can be triggered by visual stimulation, the setup is usually equipped with a projector or screen to present the visual stimulus to the fish. Although in our setups stimuli are projected below the fish, a lateral projector would be fully compatible with Stytra. Most of our rig frames consist of optomechanical parts commonly used for building microscopes. These parts are convenient but not strictly necessary to build a well-functioning rig. Replacing them with simple hardware-store and laser-cut components can significantly reduce the costs. Therefore, we also provide instructions for a head-restrained setup built inside a cardboard box, where the most expensive item is the high-speed camera, bringing the price of the whole setup without the computer below 700 euros. We built and documented such a setup, where we were able to elicit and record reliable optomotor responses in larval zebrafish (Fig 8).

A complete description of all the above-mentioned versions of the setup along with an itemized list of parts is included within the Stytra hardware documentation.

### Comparison with existing software packages

Many general-purpose systems have been proposed over the years to present visual and other kinds of stimuli and control behavioral experiments, each with its own strengths and limitations. Below we sum up some of the systems which are currently maintained, and we present how they compare to Stytra.

#### Bonsai

Bonsai [15] is a visual programming language built on top of the language C# with a reactive, dataflow-based paradigm. In Bonsai, users with little experience in programming can implement their own tracking pipelines and basic stimuli. By default Bonsai offers visualization of any data processing node, and custom visualizers. In principle, due to the generality of Bonsai, all functions of Stytra could be implemented within it. Still, implementing many features would require using a programming language uncommon in science (C#). Also, the use of several Python libraries, such as DeepLabCut, is in many cases not possible, as only a subset of Python is supported in C# through the IronPython interpreter.

#### Psychophysics toolbox

Psychophysics Toolbox [16] offers a large toolbox to build visual stimuli and stimulation protocols. The toolbox has been developed with human psychophysics in mind, in particular visual and auditory psychophysics. It provides large control over display and sound hardware, and many tools for acquiring responses from the subject through the mouse and keyboard. Still, its application is restricted to the stimulus design, as it does not offer any camera integration or animal tracking modules. This makes the toolbox ill-suited for developing closed-loop stimuli where behavior and responses of the animal need to be fed back to the stimulus control software. Moreover, it relies on the proprietary software package Matlab.

#### Psychopy

Psychopy [17] is a library similar to the Psychophysics Toolbox, written in Python. It provides precise control over displaying visual and auditory stimuli (not currently implemented in Stytra), and a set of tools for recording responses through standard computer inputs (mouse and keyboard). Due to its wide use in human psychophysics experiments, it has a larger library of stimuli than Stytra. However, it is also purely a stimulation library without video or other data acquisition support. Moreover, it does not provide a system for easy online control of stimulus parameters, an essential feature for closed-loop experiments.

#### MWorks

MWorks is a C/C++ library to control neurophysiological experiments, developed mostly for (visual) neurophysiology in primates and rodents. It provides support for building complex tasks involving trials with different possible outcomes, and contains a dedicated library for handling visual stimuli. Due to being implemented in a compiled language, higher and more consistent performance can be obtained than with our package, which is Python based. However, it is not designed for online video analysis of behavior, which is essential for behaviorally-controlled closed-loop experiments. Furthermore, while scripting and expanding Stytra requires pure Python syntax, experiments in MWorks are coded in custom high-level scripting language based on C++. Most importantly, it runs only on MacOS, which depends on Apple hardware, available only in a minority of laboratories.

#### ZebEyeTrack

The software solution described in [2] covers a small subset of Stytra functionality—eye tracking and eye-motion related stimulus presentation. It is implemented in LabView and Matlab, which adds two expensive proprietary software dependencies. Running an experiment requires launching separate programs and many manual steps as described in the publication. The tracking frame rate is limited to 30 Hz in real-time while Stytra can perform online eye tracking at 500 Hz, and Stytra’s performance is mainly limited by the camera frame rate.

## Results

### Triggering Stytra from a scanning two-photon microscope

We demonstrate the communication with a custom-built two-photon microscope. We performed two-photon calcium imaging in a seven days post fertilization (dpf), head-restrained fish larva pan-neuronally expressing the calcium indicator GCaMP6f (Tg(*elavl3*:GCaMP6f), [18]). For a complete description of the calcium imaging protocol see [19]. These and following experiments were performed in accordance with approved protocols set by the Max Planck Society and the Regierung von Oberbayern.

**Fig 9:**
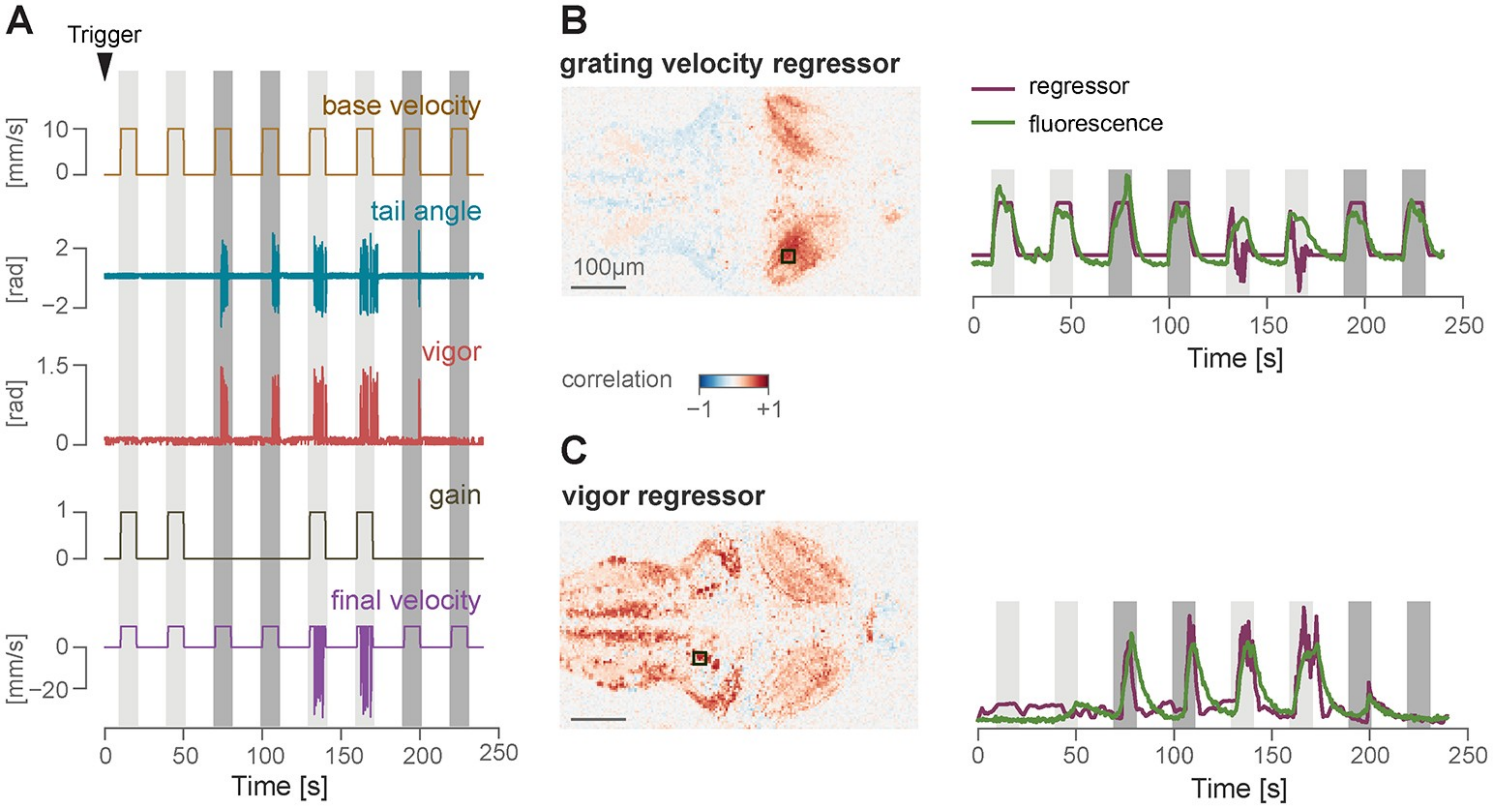
Closed-loop protocol and simultaneous whole-brain calcium imaging. A) A protocol consisting of either open- or closed-loop forward-moving gratings was presented to a seven day old Tg(*elavl3*:GCaMP6f) zebrafish larvae during two-photon imaging. The arrowhead points to the timepoint of receiving the trigger signal from the microscope. Colored stripes indicate periods when the gratings were moving: dark gray represents open loop trials (gain 0) and light gray represents closed-loop trials (gain 1). B) Left: Pixel-wise correlation coefficients with the grating velocity regressor. The square on the regressor map reports the position of the area that was used to compute the calcium trace displayed on the right. Right: z-scored fluorescence trace from the selected area, imposed over the regressor trace. C) Same as B, for the vigor regressor.

We designed a simple protocol in Stytra consisting of either open- or closed-loop forward-moving gratings, similar to the optomotor assay described in the closed-loop section, with the gain set to either 0 or 1. At the beginning of the experiment, the microscope sends a ZeroMQ message to Stytra, as described in the previous section. This triggers the beginning of the visual stimulation protocol, as well as the online tracking of the fish tail, with a 10-20 ms delay. To match behavioral quantities and stimulus features with their evoked neuronal correlates, we used the data saved by Stytra to build regressors for grating speed and tail motion (for a description of regressor-based analysis of calcium signals, see [8]). Then, we computed pixel-wise correlation coefficients of calcium activity and the two regressors. Fig 9 reports the results obtained by imaging a large area of the fish brain, covering all regions from the rhombencephalon to the optic tectum. As expected, calcium signals in the region of the optic tectum are highly correlated with motion in the visual field, while events in more caudal regions of the reticular formation are highly correlated with swimming bouts. The Stytra script used for this experiment is available at stytra/example/imaging_exp.py.

### Experiment replication

One of the main strengths of Stytra is the possibility of sharing the experimental paradigms described in a publication as scripts that can be run on different platforms and experimental hardware. To prove the validity of this approach, we decided to showcase the software reproducing the results from two publications that investigated different behaviors of the larval zebrafish. This allowed us to verify the performance of our package in producing and monitoring reliable behavioral responses, and showed how the Stytra platform can be used to share the code underlying an experimental paradigm. The scripts used for designing these experiments are available in our repository, together with a full list of parts and description of the hardware. In this way, everyone can independently replicate the experiments simply by installing and running Stytra on a suitable behavioral setup.

#### Closed-loop motor adaptation

To demonstrate the effectiveness of the closed-loop stimulation software for head-restrained larvae, we re-implemented in Stytra one of the paradigms described in [13]. This paper addresses the importance of instantaneous visual feedback in the control of the optomotor response in seven dpf zebrafish larvae.

**Fig 10:**
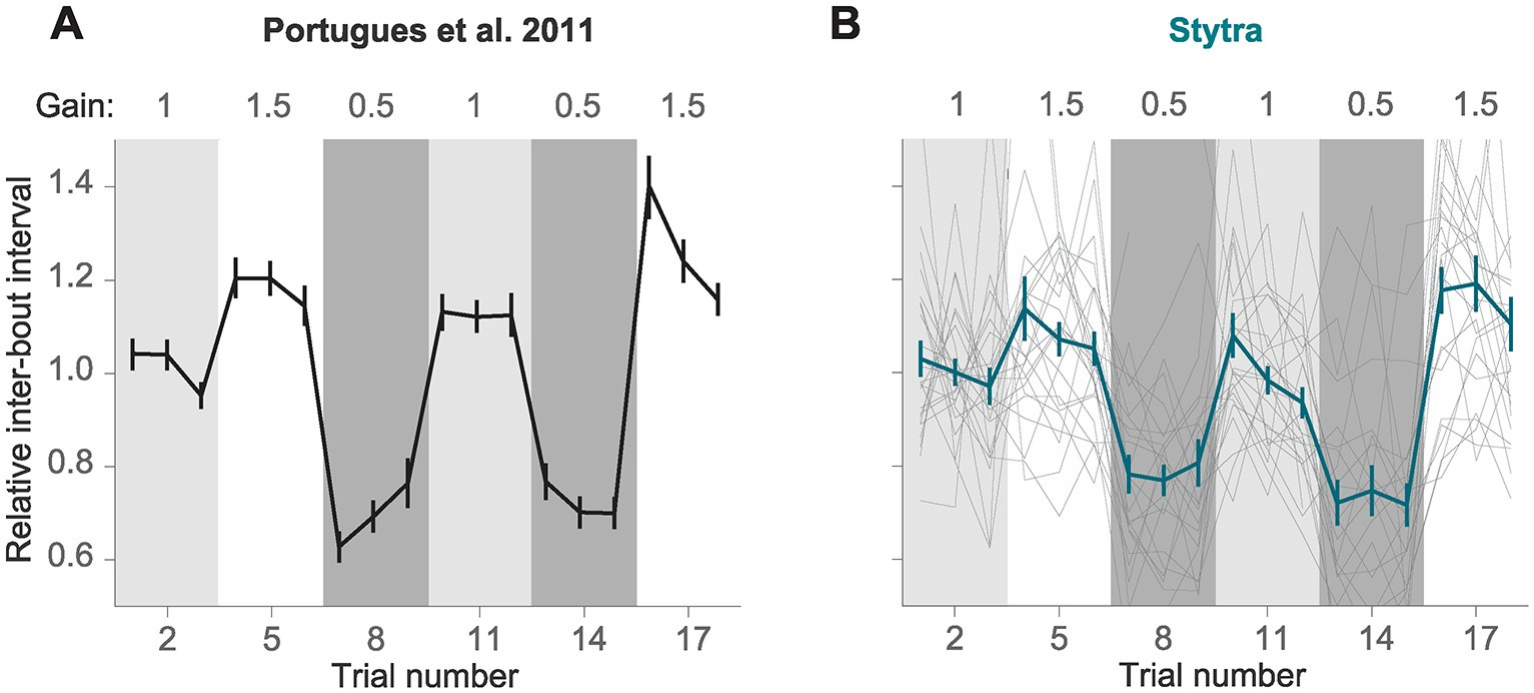
Visual feedback changes inter-bout interval in a head-restrained optomotor assay. Replication within Stytra of results published in [13]. A) Changing the gain that is used to convert the fish’s swimming vigor to relative velocity with respect to the grating affects the inter-bout interval. The line represents the average normalized inter-bout time, and bars represent standard error of the mean from n = 28 larvae (adapted from [13]). B) Replication in Stytra of the same experimental protocol (n = 24 larvae). Individual fish traces are shown in gray.

In [13], a closed-loop paradigm was used to have real-time control over the visual feedback that the animal receives upon swimming. After triggering motor activity with forward-moving black and white gratings (10 mm/s, 0.1 cycles/mm), online tail tracking was used to estimate the expected velocity of the fish based on freely-moving observations, and a backward velocity proportional to the expected forward velocity was imposed over the forward grating speed. In one crucial experiment (Fig 3 of [13]) the authors demonstrated that reducing or increasing the magnitude of this velocity by a factor of 1.5 (high gain) or 0.5 (low gain) resulted in modifications of the bout parameters such as bout length and inter-bout interval (time between two consecutive bouts). Fig 10A shows the inter-bout interval along the protocol, where the three gain conditions were presented in a sequence that tested all possible gain transitions. When the gain increased the fish was consistently swimming less (higher inter-bout interval), while the opposite was observed when the gain decreased. Therefore, as expected, fish adapted the swimming parameters to compensate for changes in visual feedback.

We reproduced exactly the same protocol within Stytra, and we used Stytra modules for closed-loop control of a visual stimulus to compare whether it could replicate the findings from [13]. The cumulative angle of the extracted tail segments was used with a gain factor to estimate the fish velocity and the gain factor was changed in a sequence matching the protocol in [13]. The replication with Stytra yielded the same result (Fig 10B), that inter-bout interval decreased in low gain conditions and increased in high gain conditions.

#### Closed-loop phototaxis assay

**Fig 11:**
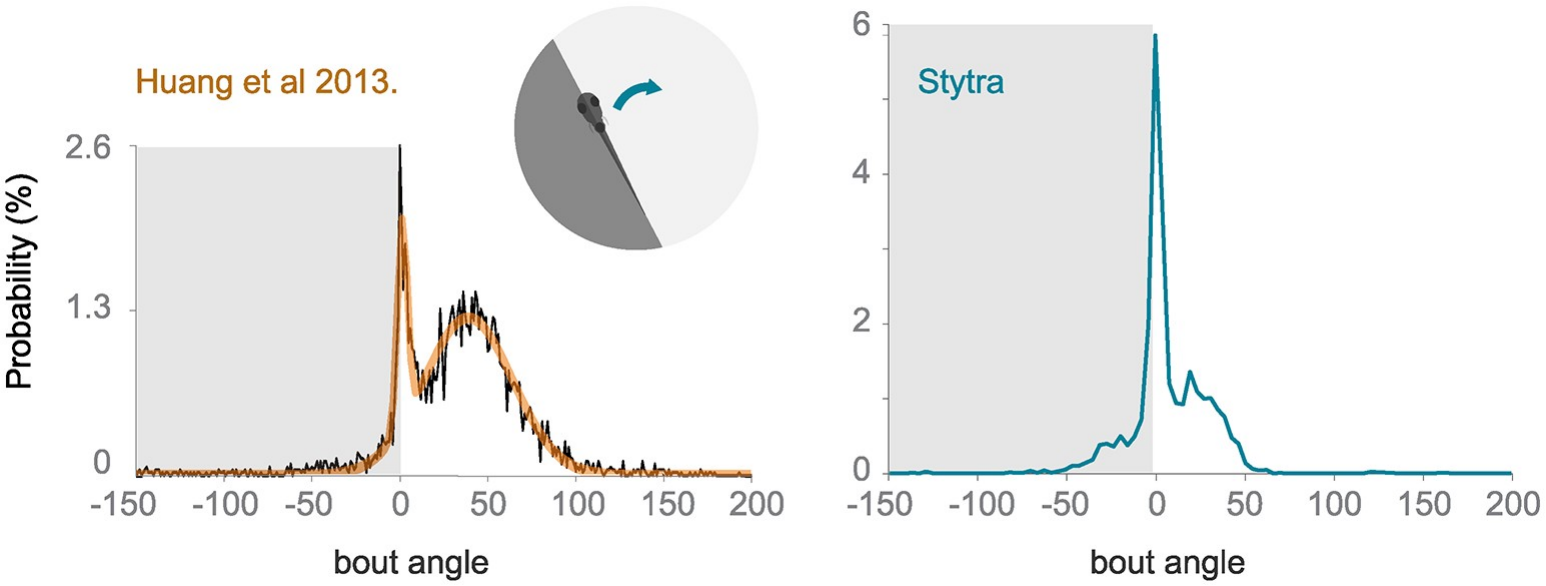
Comparison of turning angle distribution in a closed-loop freely-swimming phototaxis experiment. Left: a histogram of the angle turned per bout, redrawn from [20]. Right: the equivalent panel, with n = 10 fish and the protocol run with Stytra. The dark shading on the plot represents the dark side of the visual field.

To test the freely swimming closed-loop performance, we replicated a protocol from [20]. The fish is induced to perform phototaxis by keeping half of its visual field (the left or the right side) bright while the other is dark. The fish is more likely to turn to the bright side. The stimulus is constantly updated so that the light-dark boundary is always along the mid-line of the fish. We replicated the qualitative trends observed in [20], however the ratios of forward swims to turns are notably different (Fig 11). The variability of fish responses and differences in the stimulus presentation setup (e.g. projector brightness) could account for these differences. Also, to reduce duration of the experiments, we included a radially-inward moving stimulus that brings the fish back into the field of view.

### Discussion

We have developed Stytra, a Python-based software package that can perform online behavioral analysis and stimulation and can be interfaced with existing solutions to combine these with physiological experiments. This demonstrates its suitability as a framework for coding and running experiments in systems neuroscience. In addition to the open-source software, we are contributing to the nascent open hardware movement [4] and are providing a complete description of the hardware used for conducting behavioral experiments. Finally, we provide a set of example analysis scripts for the experiments described in this manuscript, which can be easily modified for other experimental questions. We believe that the simplicity of the implementation of an experiment within Stytra facilitates the collaboration between laboratories, since complex experimental paradigms can be run and shared with Python scripts whose reproducibility can be ensured using version control.

The current version of the software supports all experimental paradigms currently running in our lab. Support for different hardware would require some extensions in the architecture. Simultaneous use of multiple cameras is currently not supported either, but this requires a minor rewriting of the frame dispatching module. We will both continue to extend Stytra’s capabilities and support any contributions that expand the library to cover a wider range of experimental conditions. Finally, it is important to note that the choice of Python as a language would make it difficult to obtain millisecond-level or higher temporal precision (e.g. for closed-loop electrophysiology). To this aim, existing solutions based on compiled languages should be employed, such as [21] (a system for closed-loop electrophysiology in C++). Another possibility would be to combine Open Ephys and Bonsai, as in [22].

The modular and open-source nature of the package (licensed under the gnu gpl v3.0 licence) facilitates contributions from the community to support an increasing number of hardware devices and experimental conditions. Although the current implementation deals with typical zebrafish experiments, the package contains many modules that can be used in other contexts, for example: Qt-based design and timed execution of stimuli, support for different cameras models and accumulators to save data streamed from different processes that can be used for closed-loop stimuli. Although the adaptation to very different experimental conditions requires familiarity with Stytra internals, scientists interested in developing behavioral paradigms using pure Python could use many modules of Stytra as a starting point. We will make use of the community features of Github to provide assistance to any interested developers, and to support adopting the package in other labs. In conclusion, we hope that Stytra can be a resource for the neuroscience community, providing a common framework to create shareable and reproducible behavioral experiments.

### Online resources

- Stytra repository: https://github.com/portugueslab/stytra DOI:10.5281/zenodo.2548534
- Stytra documentation: http://www.portugueslab.com/stytra/
- data analysis notebooks: https://github.com/portugueslab/example_stytra_analysis
- example data from Stytra: https://zenodo.org/record/1692080
- example extension of Stytra to rat experiments: https://github.com/portugueslab/Stytra-with-DeepLabCut

## Supporting information

S1 Fig Software architecture of Stytra. A partial diagram of classes and the links between them.
(TIF)

S2 Fig Temporal jitter of a flickering stimulus. The distribution of time differences between bright-dark transitions of a stimulus set to flip between full luminosity on the red channel and darkness on every stimulus. Pure red was flashed in order to avoid artifacts of led dlp projector color multiplexing. The brightness of a small area of the display was recorded with a Ximea camera with a OnSemi python 1300 sensor at 2000 Hz.
(TIF)
