# Supplementary figures and images for: Stytra: An open-source, integrated system for stimulation, tracking and closed-loop behavioral experiments
Source: PLoS Comput Biol. 2019 Apr 8;15(4):e1006699. doi: 10.1371/journal.pcbi.1006699 (PMC6472806; doi:10.1371/journal.pcbi.1006699)

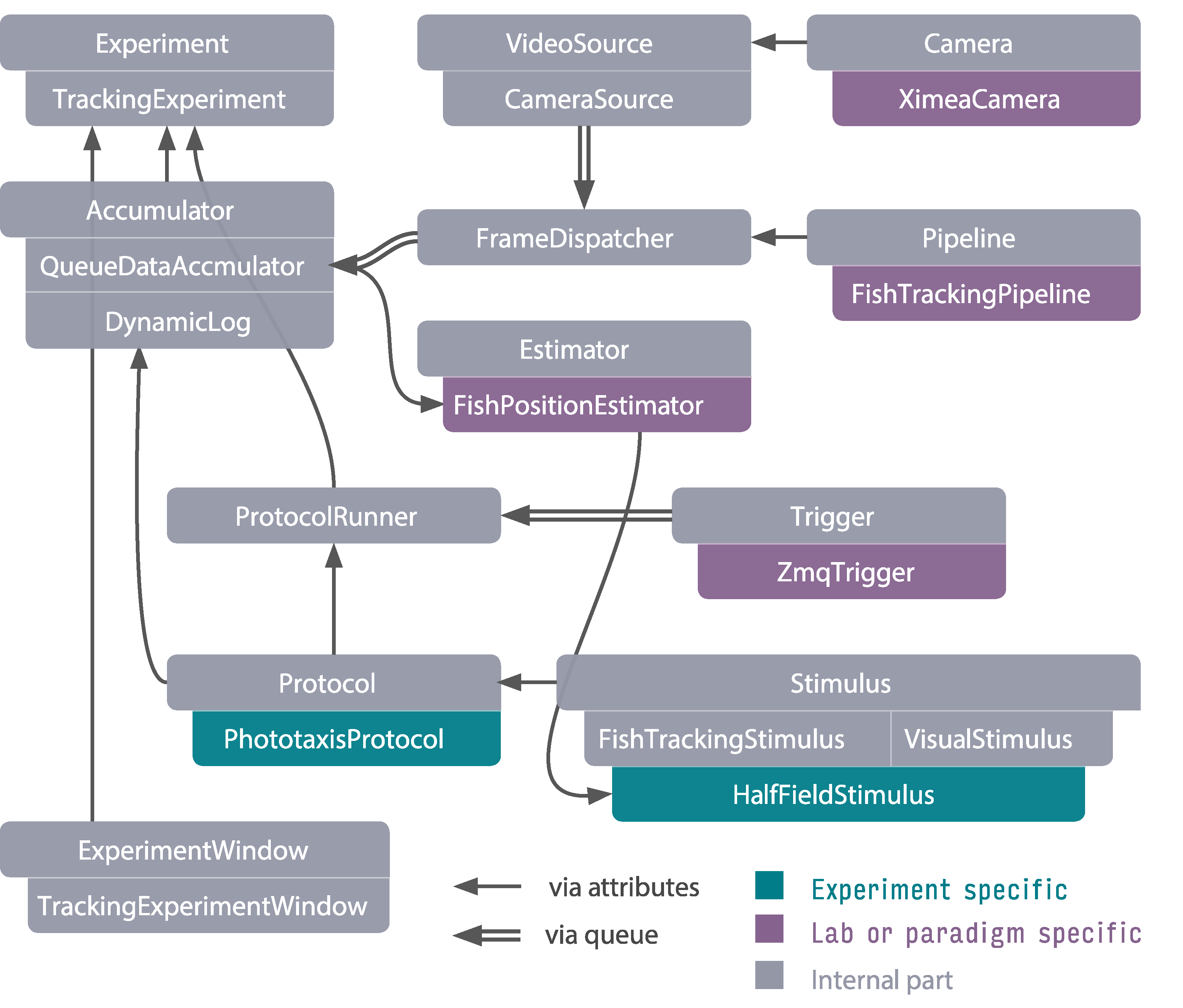

Supplement: S1 Fig — A partial diagram of classes and the links between them. (TIF) [file pcbi.1006699.s001.tif]

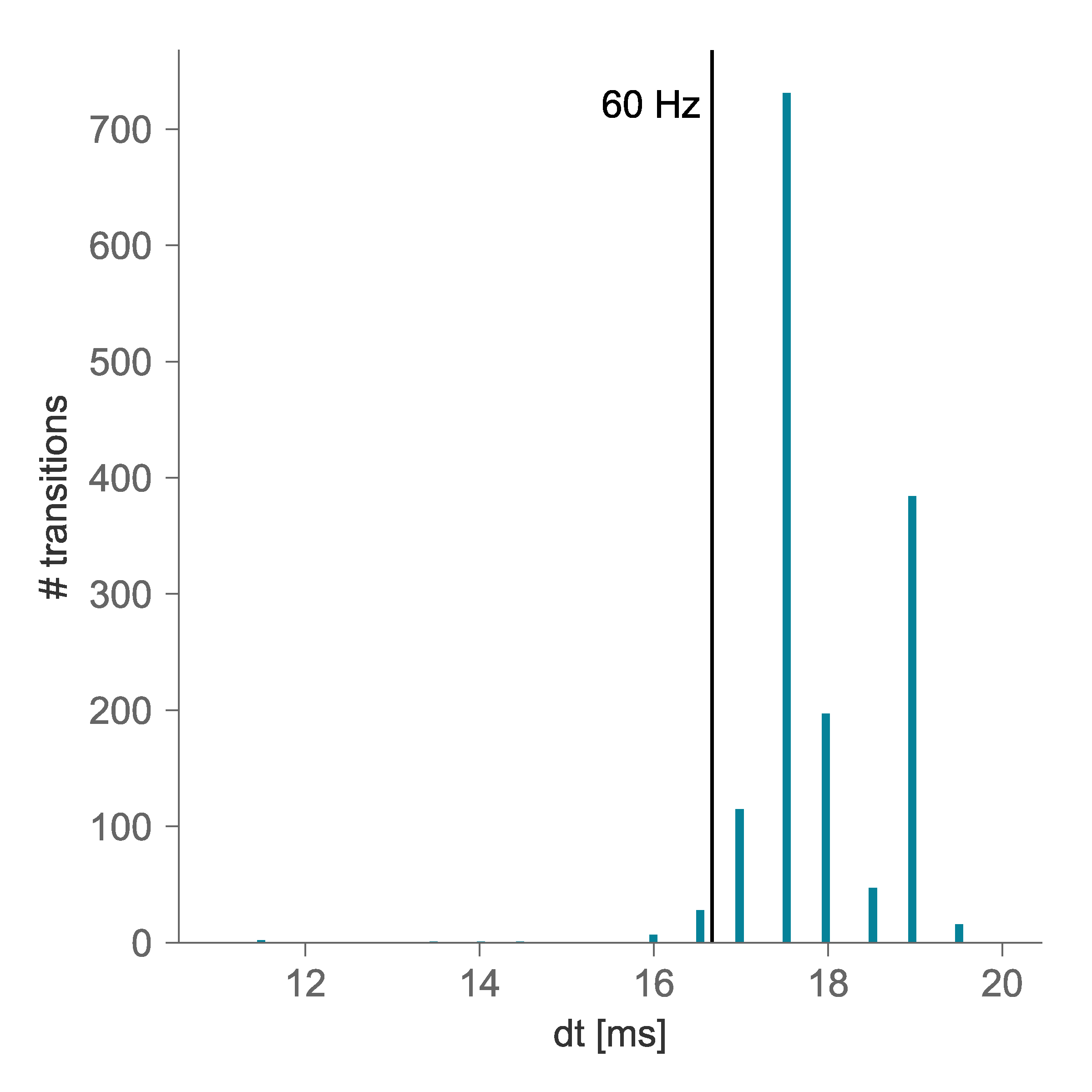

Supplement: S2 Fig — The distribution of time differences between bright-dark transitions of a stimulus set to flip between full luminosity on the red channel and darkness on every stimulus. Pure red was flashed in order to avoid artifacts of led dlp projector color multiplexing. The brightness of a small area of the display was recorded with a Ximea camera with a OnSemi python 1300 sensor at 2000 Hz. (TIF) [file pcbi.1006699.s002.tif]
